# Factors Associated with Stunting among Children under 5 Years in Five South Asian Countries (2014–2018): Analysis of Demographic Health Surveys

**DOI:** 10.3390/nu12123875

**Published:** 2020-12-18

**Authors:** Nidhi Wali, Kingsley E. Agho, Andre M.N. Renzaho

**Affiliations:** 1 School of Social Sciences, Western Sydney University, Locked Bag 1797, Penrith, NSW 2751, Australia; Andre.Renzaho@westernsydney.edu.au; 2 School of Health Sciences, Western Sydney University, Campbelltown Campus, Locked Bag 1797, Penrith, NSW 2571, Australia; K.Agho@westernsydney.edu.au; 3 African Vision Research Institute, University of KwaZulu-Natal, Westville Campus, Durban 3629, South Africa; 4 Translational Health Research Institute, Western Sydney University, Locked Bag 1797, Penrith, NSW 2571, Australia; 5 Maternal, Child and Adolescent Health Program, Burnet Institute, Melbourne 3004, Australia

**Keywords:** child undernutrition, factors, infants, stunting, South Asia

## Abstract

South Asia continues to be the global hub for child undernutrition with 35% of children still stunted in 2017. This paper aimed to identify factors associated with stunting among children aged 0–23 months, 24–59 months, and 0–59 months in South Asia. A weighted sample of 564,518 children aged 0–59 months from the most recent Demographic and Health Surveys (2014–2018) was combined of five countries in South Asia. Multiple logistic regression analyses that adjusted for clustering and sampling weights were used to examine associated factors. The common factors associated with stunting in three age groups were mothers with no schooling ([adjusted odds ratio (AOR) for 0–23 months = 1.65; 95% CI: (1.29, 2.13)]; [AOR for 24–59 months = AOR = 1.46; 95% CI: (1.27, 1. 69)] and [AOR for 0–59 months = AOR = 1.59; 95% CI: (1.34, 1. 88)]) and maternal short stature (height < 150 cm) ([AOR for 0–23 months = 2.00; 95% CI: (1.51, 2.65)]; [AOR for 24–59 months = 3.63; 95% CI: (2.87, 4.60)] and [AOR for 0–59 months = 2.87; 95% CI: (2.37, 3.48)]). Study findings suggest the need for a balanced and integrated nutrition strategy that incorporates nutrition-specific and nutrition-sensitive interventions with an increased focus on interventions for children aged 24–59 months.

## 1. Introduction

Inadequate nutritional status of children is a significant public health problem in low and middle-income regions (LMICs) around the world [1]. Recent estimates from the Global Burden of Diseases indicate that, globally, stunting declined from 36.9% in 2000 to 26.6% in 2017 [2]. However, despite the observed improvements in nearly all LMICs, South Asia (SA), along with sub-Saharan Africa and Central Asia, continue to have the highest prevalence of stunting [2]. The prevalence of stunting in South Asia although declined from 51% (89 million children) in 2000 to 35% (59 million children) in 2017 [3], it is still very high by international standards (well above the prevalence threshold of 30%—a trigger-level as a basis of public health decisions) [4]. Stunting affects immediate growth and development of children with long-term effects as adults [5,6]. Stunting is associated with poor school performance in children and lower work productivity as adults. Stunting in children is associated with increased likelihood of being overweight and chronic diseases such as type 2 diabetes, cardiovascular disease, diabetes and cancer, and mental health issues later in life [6,7,8].

Several studies have examined factors associated with stunting in the region highlighting these dimensions as complex and multi-dimensional at the individual, household, community and national levels [9]. Research reveals that the most common factors associated with stunting are individual maternal factors (education, short stature, poor nutrition health before and during pregnancy) [10,11,12,13,14,15], child factors (illness, age, inappropriate feeding practices including poor diet during the 1000 days of life and illness), household factors (household wealth index, family size and place of residence) [5,6,16,17,18,19] and access and utilisation of services (health service utilisation, and water and sanitation) [10]. However, studies have been selective in factors examined, hence making it difficult to generalise findings. Secondly, most of the studies in the region have predominantly focussed on children aged <24 months as stunting has been linked to inadequate nutrition and repeated bouts of infection during the first 1000 days of a child’s life. However, recent studies in SA suggest that the risk of stunting among preschool children increases with age, with older children (12–59 months) more likely to be stunted than their younger counterparts [20,21,22].

Recent Demographic Health Surveys (DHS) indicate that stunting rates among SA children aged 24–59 months were significantly higher than those children aged 0–23 months. For example, stunting rates among five SA countries in children aged 0–23 months lie between 27% and 28% whereas, stunting rates among children aged 24–59 months ranged from 41% to 44%. Understanding factors associated with stunting in older preschool children is critical to address the long-term effect of stunting later in life [6,7,8]. Lastly, most of these studies have been country-specific except a population-based cross-sectional study conducted in five SA countries, which looked at children below 2 years of age but does not examine stunting in children between 24–59 months and 0–59 months [23]. Our study builds upon these limitations by pooling data from five SA countries using the most recent DHS datasets (2014–2018). By pooling DHS data that include all ages of children aged <5 years, our analysis permits enhanced statistical power to address inconsistencies in the current evidence to identify sources of diversity across various DHS datasets in the region and to compare outcomes models across settings. This study aims to understand factors most significantly associated with child stunting, and severe stunting for children aged <5 years in three age groups: 0–59 months, 0–23 months and 24–59 months. It is the first pooled analysis to understand factors associated with child stunting in all ages of children aged <5 years in South Asia.

Evidence from the pooled analysis will enable public health researchers to identify common interventions for stunting that may be effective for subsets of children with common dietary patterns as these populations share similar challenges of poor public services, poverty, and gender inequality [24]. This will enable to understand factors associated with stunting for all children aged >5 years and generate evidence to inform future policy and practice. Additionally, country-specific findings were also reported to inform the core intervention components needed to reduce stunting in each South Asian country by 2030.

## 2. Materials and Methods

This study is based on analysis of existing datasets in the Demographic Health Survey (DHS) repository that are freely available online with all identifier information removed. It utilised datasets from the most recent 2014–2018 DHS conducted within 5 South Asia countries including Bangladesh, India, Nepal, Maldives, and Pakistan. Data for other South Asian countries were not available through DHS due to the following reasons: Afghanistan does not collect anthropometric data for children under 5 years of age, data for Bhutan are unavailable on DHS and finally, data for Sri Lanka have restricted access and are not publicly available for research purposes. Data were obtained from a password-enabled DHS website [25]. The DHS data were nationally representative and population-based surveys, collected by country-specific ministries of health or other relevant government-owned agencies, with technical support largely provided by Inner City Fund (ICF) International. These surveys were comparable, given the standardised nature of the data collection methods and instruments [26]. The datasets were pooled to ascertain the most significant factors associated with child stunting and severe stunting across the South Asian countries.

### 2.1. Data Sources

The DHS is a nationally representative survey that collects data on the health status of people, including reproductive health, maternal and child health, mortality, nutrition, and self-reported health behaviour among adults [26]. Information was collected from eligible women, that is, all women aged 15–49 years who were either permanent residents in the households or visitors present in the households on the night before the survey. Child health information was collected from the mother based on the youngest child aged less than five years, with response rates that ranged from 96% to 99%. Detailed information on the sampling design and questionnaire used is provided in the respective country-specific Measure DHS reports [25]. Our analyses were restricted to 564,518 children aged 0–59 months for 5 South Asian countries.

### 2.2. Study Variables

The outcome variable was stunting (height-for-age). Stunting is an indicator of linear growth retardation and cumulative growth deficits in children. The height-for-age *Z*-score (HAZ), as defined according to 2007 WHO growth reference, expresses a child’s height in terms of the number of standard deviations (SD) above or below the median height of healthy children in the same age group or in a reference group. This study focused on children with a height-for-age *Z*-score below minus two standard deviations (−2 SD) as stunted and height-for-age *Z*-score below minus three standard deviations (−3 SD) as severely stunted. Prior to computing the prevalence and further analyses were undertaken, biologically implausible values (HAZ < −6 SD or > 6 SD) were excluded [27].

### 2.3. Potential Confounding Factors

The choice of confounding factors used in this study was informed by the UNICEF framework [9]. The framework includes immediate factors including individual-level factors of diet and disease occurrence, underlying factors including household factors, and basic factors such as place of residence and country.

The confounding factors were organised into five groups: (i) Immediate factors: dietary diversity score and child’s disease occurrence (episodes of diarrhoea and fever in the last two weeks), feeding practices such as currently breastfeeding and duration of breastfeeding, Vitamin A supplementation, Vaccination coverage, child’s age and sex; underlying factors (ii) Mother’s characteristics: such as age, age at birth, height, BMI, marital status, birth order and interval, maternal and paternal education, women’s power over household earnings, household decision-making and health care autonomy, (iii) Household factors: Pooled household wealth index, access to source of water and type of toilet which were categorised into improved and unimproved sources, (iv) Access and utilisation of services: Healthcare utilisation factors such as place and mode of delivery, combined birth rank (the position of the youngest under-five child in the family), and birth interval (the interval between births; that is, whether there were no previous births, birth less 24 months prior, or birth more than or equal to 24 months prior), delivery assistance, antenatal clinic visits (ANC) and access to media services, listening to the radio, watching television, and reading newspapers or magazines; (v) Basic factors such as country and place of residence (urban or rural). In order to reduce collinearity, we combined place of birth and mode of delivery and, birth order and birth interval. The combined mode of delivery and place of birth was divided into three categories as delivered at home, delivered at a health facility with non-caesarean section and delivered at a health facility with a caesarean section while, the combined birth order and the birth interval was classified as birth rank and birth interval, which is consistent with previous studies [28]. Maternal height was divided in the 5 following categories: <145 cm, 145–149.9 cm, 150–154.9 cm, 155–159.9 cm, and ≥160 cm, with <145 cm defined as short maternal height [29].

The household wealth index for the pooled dataset was constructed using the “hv271” variable. The hv271 variable used that principal components statistical procedure which was used to determine the weights for the wealth index based on information collected about 22 household assets and facilities and produce the standardised scores (z-scores) and factor coefficient scores (factor loadings) of wealth indicators. In the household wealth index categories, the bottom 20% of households were arbitrarily referred to as the poorest households, and the top 20% as the richest households, and was divided into poorest, poor, middle, rich, and richest.

Dietary diversity (DD) was calculated by summing the 7 food groups consumed during the last 24 h. These foods are grains roots and tubers, legumes and nuts, Milk/dairy products, flesh foods (meat, fish, poultry and liver/organ meats), vitamin-A rich fruits and vegetables other fruits and vegetables and eggs, and were categorised into two groups, namely, the child had ≥4 food groups and the child had <4 food groups [30].

### 2.4. Statistical Analysis

To examine factors associated with stunting among children aged 0–23 months, 24–59 months and children 0–59 months, the dependent variables were expressed as a binary outcome, i.e., category 1 [stunted (≥2 SD) or severely stunted (≥3 SD)]. For the combined 5 South Asian countries, a population-level weight, unique country-specific clustering, and strata were created to avoid the effect of countries with a large population (such as India with over 1.4 billion people in 2017 [31] offsetting countries with a small population (such as the Maldives with about 437,535 people in 2017 [32]. Population-level weights were used for survey (SVY) tabulation that adjusted for a unique country-specific stratum, and clustering was used to determine the percentage, frequency count and estimating the rates and 95% confidence intervals of child stunting in each country.

**Figure 1:**
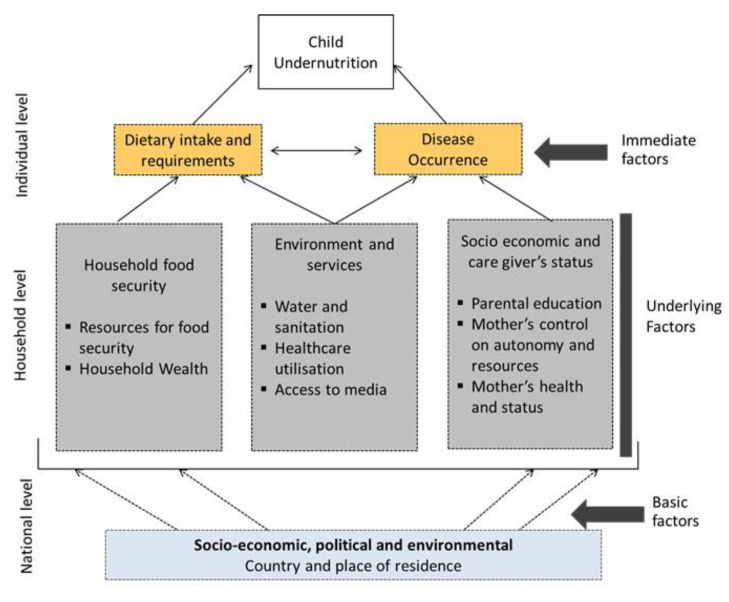
Conceptual framework of the determinants of child undernutrition.

Using three stages as described in Figure 1, the associations were further tested by odds ratios (OR) using univariate survey logistic regression analyses, and then hierarchical multiple survey logistic regression analyses. In the first stage model, basic factors were entered into the model. In the second stage model, underlying factors were added to the basic factors. A similar procedure was employed for the third stage model, which included the basic, underlying factors, as well as access to immediate factors. The aim of this hierarchical multiple logistic regression analyses was to allow for a comparison of the relationship between each of the different sets of factors described in Figure 1 in examining factors associated with stunting among children under 5 years. All statistical analyses were conducted using STATA/MP Version.14.1 (StataCorp, College Station, TX, USA) and adjusted odds ratios (AORs) and their 95% confidence intervals (CIs) obtained from the adjusted hierarchical multiple logistic regression model were used to measure the factors associated with child stunting.

## 3. Results

### 3.1. Prevalence of Stunting in Children Aged 0–23 Months, 24–59 Months and 0–59 Months

**Figure 2:**
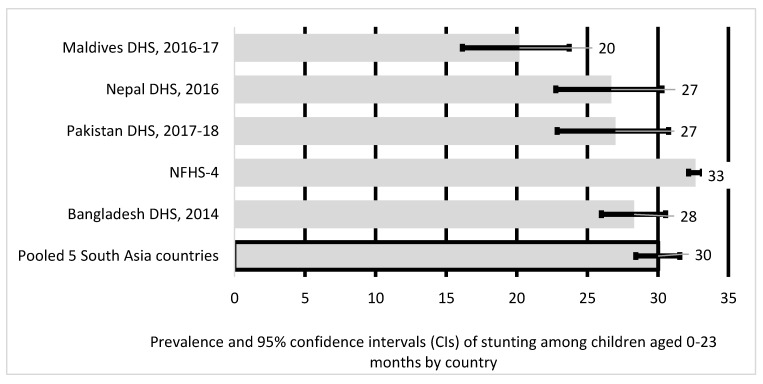
Prevalence and 95% confidence intervals of stunting in children aged 0–23 months in South Asia.

As illustrated in Figure 2, the prevalence of stunted children aged 0–23 months was highest in India (33%) and lowest in the Maldives (20%). The overall pooled prevalence of stunted children aged 0–23 months in five South Asian countries was 30%.

**Figure 3:**
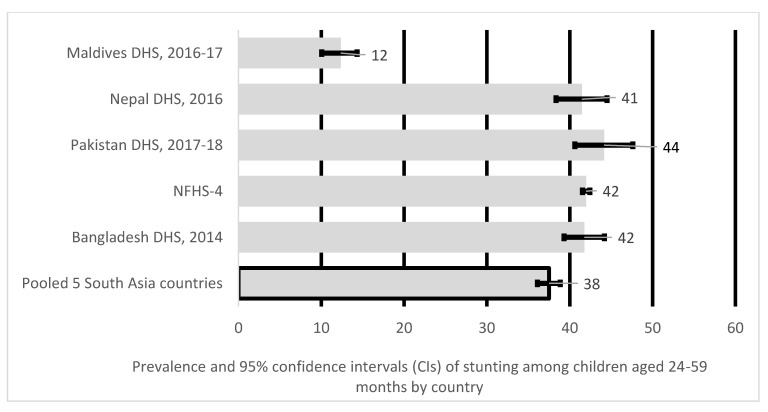
Prevalence and 95% confidence intervals (CIs) of stunting in children aged 24–59 months in South Asia.

As indicated in Figure 3, the pooled prevalence of stunting among children aged 24–59 months in five South Asian countries was 38%, with Pakistan reporting the highest prevalence of stunting at 44%, 42% in Bangladesh and India, and lowest at 12% in the Maldives.

**Figure 4:**
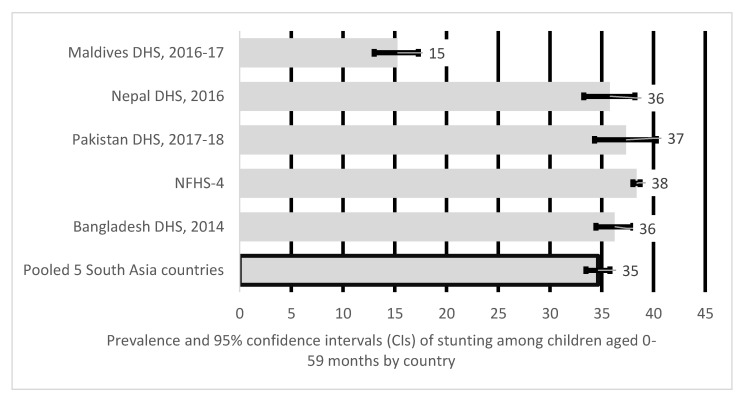
Prevalence and 95% confidence intervals of stunting in children aged 0–59 months in South Asia.

The pooled prevalence of stunting in children aged 0–59 months in South Asia (Figure 4) was 35%. India had the highest prevalence (38%) of stunting amongst children aged 0–59 months. Prevalence in Pakistan was at 37%, Bangladesh and Nepal at 36%, the Maldives the lowest at 15%.

Supplementary material 1 provides the characteristics of the sample of parents and children aged 0-59 months in five South Asian countries. 

### 3.2. Factors Associated with Child Stunting for Children Aged 0–23 Months

Table 1 showed the factors associated with stunting among children aged 0–23 months. Mothers with no education [AOR = 1.65; 95% CI: (1.29, 2.13)] and maternal short stature (<150 cm) [AOR = 2.00; 95% CI: (1.51, 2.65)] were common factors stunting across all ages of children aged <5 years. Children of formerly married mothers [AOR = 0.37; 95% CI: (0.15, 0.92)] were less likely to be stunted than those of currently married mothers. Other factors associated with stunting among children aged 0–23 months were: children born through vaginal birth at a facility [AOR = 1.61; 95% CI: (1.30, 2.00)] and those born at home [AOR = 1.81; 95% CI: (1.42, 2.31)] were at higher odds of stunting. Children over 12 months had increased odds of stunting and male children were more likely to be stunted than female children [AOR = 1.22; 95% CI: (1.08, 1.39)]. Country-level data revealed maternal short stature (<150 cm), mothers with no education and children over the age of 12 months were common factors for child stunting across all 5 South Asian countries (see supplementary material 2).

### 3.3. Factors Associated with Child Stunting for Children Aged 24–59 Months

Table 2 reported factors associated with stunting among children aged 24–59 months. Children aged 24–59 months were more likely to be stunted if they lived in India [AOR = 2.69; 95% CI: (1.77, 4.08)], Nepal [AOR = 2.62; 95% CI: (1.71, 4.02)] and Pakistan [AOR = 3.28; 95% CI: (2.09, 5.15)]. Children of mothers with no education [AOR = 1.46; 95% CI: (1.27, 1. 69)], BMI <= 18.5 [AOR = 1.42; 95% CI: (1.16, 1. 73)], shorter than 150 cm [AOR = 3.63; 95% CI: (2.87, 4.60)], attended less than 3 ANC visits [AOR = 1.41; 95% CI: (1.05, 1.88)], belonged to poorer households [AOR = 1.43; 95% CI: (1.17, 1.74)], were 2nd/3rd birth order with less than or equal to 2 years interval [AOR = 1.37; 95% CI: (1.16,1.61)] were more likely to be stunted. Children of mothers watched television [AOR = 0.86; 95% CI: (0.76, 0.97)] were at lower odds of being stunted (see Table 3 for details). Country-level data revealed maternal short stature (<150 cm) was the common factor for child stunting across all 5 South Asian countries (see supplementary material 2).

### 3.4. Factors Associated with Child Stunting for Children Aged 0–59 Months

Table 3 reported factors associated with stunting among children aged 0–59 months. Children aged 0–59 months were more likely to be stunted if they lived in India [AOR = 2.07; 95% CI: (1.44, 2.98)], Nepal [AOR = 1.77; 95% CI: (1.25, 2.51)] and Pakistan [AOR = 2.28; 95% CI: (1.57, 3.31)]. Factors significantly associated for stunting in this age group were mothers with no education [AOR = 1.59; 95% CI: (1.34, 1.88)], mother’s short stature <150 cm [AOR = 2.87; 95% CI: (2.37, 3.48)], BMI <= 18.5 [AOR = 1.36; 95% CI: (1.14, 1.63)], children born at home [AOR = 1.43; 95% CI: (1.11, 1.84)], attended less than 3 ANC visits [AOR = 1.35; 95% CI: (1.09, 1.67)], belonged to poorest households [AOR = 1.39; 95% CI: (1.13, 1.73)], were 2nd/3rd birth order with less than or equal to 2 years interval [AOR = 1.19; 95% CI: (1.07, 1.31)] were more likely to be stunted. Children aged 24–59 months had increased odds of stunting and male children were more likely to be stunted than female children [AOR = 1.05; 95% CI: (0.96, 1.14)]. Country-level data revealed maternal short stature (<150 cm) was the common factor for child stunting across all 5 South Asian countries (see supplementary material 2).

## 4. Discussion

Our results indicate overall stunting prevalence in five South Asia countries for the study period (2014–2018) were 30% for children aged 0–23 months, 38% for children aged 24–59 months and 35% for children aged 0–59 months. For infants between 0–23 months, India had the highest stunting levels at 33% and Maldives the lowest at 20%, while for children between 24–59 months prevalence was much higher above 40% in most South Asian countries except for Maldives at 12%, with over one-third of South Asian children <5 years stunted. The stunting prevalence in the Maldives, although lowest amongst other South Asian countries, is still high for a low- and middle-income countries (LMICs). The stunting rate reported in this study was higher than the projected Global stunting (20.8%) in 2020 for children aged 0–59 months [33]. The stunting prevalence across most South Asian countries was above 30% threshold, making it a critical public health issue in the region [4]. We also noted uneven stunting prevalence amongst the 5 South Asian countries. Our research finds that children <5 years of age were more likely to be stunted if they lived in India, and Pakistan, while prevalence was higher within those aged 24–59 months living in India, Nepal and Pakistan when compared to younger children aged 0–23 months. The variation in the rates of stunting amongst the 5 South Asian countries may be due to health inequities and inequalities, as well as social, economic and cultural issues within and across different populations [34]. However, further research is required to understand these variations amongst the South Asian countries.

Literature in South Asia has mainly focused on the 1000 days of conception to two years of a child’s life [5,6,16,17,18,19]. While it is important to focus on the first two years of a child’s life, our findings suggest children aged 24–59 months were more likely to be stunted than their younger counterparts (0–23 months). Recent studies in LMICs including South Asian countries of Bangladesh, India and Nepal concur that stunting prevalence increases with child’s age [35,36,37]. This may be explained by the effect of breastfeeding transition where most children are breastfed until 24 months; breastfeeding gradually declines with child’s age coupled with poor or limited complementary feeding and dietary diversity. Shorter birth intervals between children can also lead to more attention given to the newborn, leaving older children with unattended needs. Our research found that children aged 24–59 months and 0–59 months had higher odds of being stunted if they were born second or within an interval less than or equal to two years. There is growing evidence that suggests when pregnancies and births are spaced closely, women may not have the opportunity to replenish their nutritional stores stressed during pregnancy and breastfeeding and might give poor attention to children’s needs, resulting in adverse health outcomes in children such as stunting [38]. Longer birth intervals of more than 3 years may also provide protection against child stunting [39,40]. Our study highlights the need for increased attention in research and interventions on child’s nutrition for all children aged <5 years, especially those children aged 24–59 months of age, where the prevalence was much higher than the 30% threshold. This increased focus will ensure South Asian countries meet the Sustainable Development Goal 2.2 of eliminating child stunting by 2030 [41].

Our findings revealed that children aged 0–23 months of formerly married mothers were less likely to be stunted than those of currently married mothers. Further analysis from our study indicated that about 71% of formerly married mothers had secondary or higher education compared to 29% with primary or no education. Overall, mother’s education was significantly associated with stunting among all children aged <5 years. This finding is consistent with previous studies conducted in South Asian countries of Pakistan [42] and Bangladesh [43] and other LMICs of Kenya [44] and Indonesia [45]. A nationally representative survey conducted in Afghanistan, Bangladesh, India, Nepal and Pakistan found that mother’s education was a strong predictor for optimal breastfeeding practices including early initiation of breastfeeding (EIBF), whereas women with low autonomy were significantly associated with suboptimal breastfeeding practices including EIBF [46]. Women’s education has been linked with extended life expectancies, reduced mortality and overall improved child health and nutrition [47,48,49]. This can be through a better understanding of treatment and prevention of health services and enhanced health-seeking behaviours. It can also contribute to delayed childbearing, longer birth intervals and having fewer children, all of which can positively impact overall health outcomes in children [50,51].

Short maternal stature (<150 cm) was associated with stunting among all children aged <5 years. A research conducted in 35 LMICs found the association of shorter maternal height with stunting among children aged 12–59 months [29]. Other research amongst children aged 6–23 month [52] and 0–59 month [23] showed similar associations. Maternal height provides a useful marker for assessing intergenerational linkages in child’s health before or immediately after birth with lasting influence over a few years. This could be due to the genetic background as well as environmental factors such as diet, nutrition and culture that impact the mothers during early childhood and then the growth of their child [23,29]. Lower maternal BMI (≤18.5 kg/m^2^) was associated with higher odds of stunting in children aged 24–59 months and 0–59 months. This finding is consistent with research conducted in 35 LMICs using cross-sectional data, that found stunting was higher among children aged 12–59 months with mothers with BMI < 18.5 as compared to those with BMI ≥ 25.0 [29]. This study highlights foetal origins of childhood undernutrition as intrauterine intergenerational transmission of low maternal BMI during pregnancy giving infants high risk of low birth weight and being small for gestational age. Our study further points that this association is statistically more significant for children aged 24–59 months when compared to younger children (0–23 months), which further highlights the need for increased attention in policy and program for children aged 24–59 months.

Children aged 24–59 months and 0–59 months belonging to poorer households were at higher odds of being stunted when compared to those from wealthy households. This finding is similar to previous research conducted in Bangladesh [53], India [54] and Indonesia [55]. Additional analysis from a univariate analysis indicated that children aged 0–23 months from poorest households were 2.45 times [OR = 2.45, 95% CI (1.99–3.02)] more likely to be stunted than those from richest households but after adjusting for underlying factors, household wealth index was not associated with stunting. The finding was in contrary to previous research [56].

Our findings suggest that male children were more likely to be stunted than female children, especially for children aged 0–23 months. This finding is consistent with research in LMICs [57,58] but contrary to prior studies in South Asia [59,60,61]. Research in South Asia highlights female children are more likely to be undernourished as compared to male children. This finding may be attributed to the family’s preference or desire to have a male child over a female child which often results in neglect of female children and further explains the higher child survival rates for the male child as compared to females in the region [59,60,61]. Changing dietary patterns where increased consumption of unhealthy and sugary foods in South Asia [62,63] might be preferred for the male child over female, as they could be viewed as a sign of wealth for families but have adverse nutrition outcomes.

Children of mothers who took less than three or no antenatal care visits to the health institution prior to childbirth showed higher odds of stunting. Higher coverage of ANC (4 + ANC visits) has shown significant impacts on improved stunting outcomes in South Asian countries of Afghanistan, Bangladesh, India, Nepal, and Pakistan [23] and also Indonesia [55] and Tanzania [64]. This knowledge has led to increased policy efforts across South Asia to improve access and coverage of maternity services, including antenatal and postnatal care and accessing health institutions [65]. Despite the increased efforts, children continue to be delivered at home in South Asia. Our research found children aged 0–23 months and 0–59 months, who were delivered at home were at higher odds of being stunted when compared to those born in a health facility. This finding is consistent with studies in India, Indonesia and Sub-Saharan Africa [66,67,68].

Children delivered with the help of traditional birth attendants (TBAs) were less likely to be stunted than those delivered by a health professional. This difference could be attributed to the fact that in rural South Asia, TBAs play an important role in the health care systems by supporting Infant and Young child feeding practices (breastfeeding and complementary feeding) and providing advice on several health-related topics to all mothers, especially young and inexperienced mothers [69,70,71].

Children aged 0–23 months who had adequate diverse food (4+ foods) were 20% less likely to stunted as compared to those children with inadequate dietary diversity (<4 foods). This association was consistent with a cross-sectional study conducted in 14 LMICs, which found that the consumption of a minimum acceptable diet with dietary diversity and adequate intake of iron-rich foods reduced the risk of stunting [35]. However, dietary diversity was not a significant factor for stunting for children aged 2–5 years. This can be explained as children are mostly breastfed for the first 6 months with adequate attention given to their diet during the breastfeeding transition until 24 months, but breastfeeding gradually declines with child’s age coupled with poor or limited complementary feeding and dietary diversity with increased consumption of unhealthy food and sugary drinks with often resulting in poor nutritional outcomes [63].

Our findings revealed that at basic and underlying factors (Model 2), children aged 0–59 months who lived in rural areas were more likely to be stunted than those that lived in urban areas. Type of residence did not remain significant when exposed to other immediate variables. This finding is consistent with a previous cross-sectional study conducted in Pakistan, which found increased stunting in rural areas in the univariate analysis [20].

This study had several strengths and limitations. One of the strengths of the study was to examine factors associated with stunting among all children aged <5 years, across three age groups: 0–59 months, 0–23 months and 24–59 months. The study was population-based with a high response rate of an average of 97% for children and utilised the most recent nationally representative data in the five countries in South Asia. Despite these strengths, this study has limitations worth highlighting. Firstly, as cross-sectional data were used to identify the factors of stunting the study was unable to establish a causal relationship between the study characteristics and child stunting. Secondly recall and measurement errors may have overestimated or underestimated the findings of this study, as data regarding some of the study factors were obtained from mothers up to five years after childbirth. Lastly, DHS does not capture data on unhealthy food and sugary drink consumption for children aged 2–5 years that might be critical to understand its association with child nutrition including stunting and obesity.

## 5. Conclusions

This study indicated mother’s education (no education) and maternal height were the most significant factors associated with stunting. Children born to mothers with BMI <= 18.5, those born through a vaginal birth and at home, living in India, Nepal and Pakistan, poor dietary diversity, child’s age and gender also determined child stunting across South Asian countries. Additionally, our findings suggest that male children were more likely to be stunted than female children. Our findings suggest the need for a balanced and integrated nutrition strategy that incorporates nutrition-specific interventions (focus on immediate factors) along with nutrition-sensitive interventions (focus on underlying factors) implemented across the region. Nutrition-specific programs include vitamin A and zinc supplementation, exclusive breastfeeding, dietary diversity promotion and food fortification, whilst nutrition-sensitive interventions influence the underlying determinants of nutrition notably maternal education, Water Sanitation and Hygiene (WASH); child protection; schooling; early child development; maternal mental health; agriculture and food security; health and family planning services; social safety nets; and women’s empowerment. It is also crucial that these interventions are targeted at all children < 5 years of age. At present, while there are interventions targeted for 0–23 months during the first 1000 days of a child’s life, there needs to be an increased focus of interventions for children aged 24–59 months. Our study generates evidence to this effect and stresses the need for timely initiation of supplementary feeding to meet the growing nutritional requirements of preschool children.

## Figures and Tables

**Table 1:** Factors associated with stunting in children aged 0–23 months.

Variables	Model 1: Basic				Model 2: Basic and Underlying				Model 3: Basic, Underlying and Immediate			
	OR	95% CI		*p*-Value	OR	95% CI		*p*-Value	OR	95% CI		*p*-Value
**Countries**												
Maldives	1.00				1.00				1.00			
India	1.83	1.11	3.02	0.018	1.74	0.99	3.08	0.055	1.41	0.78	2.57	0.250
Bangladesh	1.73	1.02	2.93	0.042	1.41	0.78	2.57	0.255	1.12	0.60	2.06	0.727
Nepal	1.74	0.99	3.09	0.056	1.14	0.62	2.11	0.678	0.87	0.46	1.66	0.682
Pakistan	1.51	0.85	2.67	0.160	1.50	0.80	2.82	0.207	1.29	0.67	2.50	0.447
**Type of place of residence**												
Urban	1.00				1.00				1.00			
Rural	1.34	1.12	1.61	0.001	1.02	0.86	1.22	0.808	1.01	0.86	1.19	0.891
**Working status**												
Not working					1.00				1.00			
Working					1.35	1.00	1.82	0.052	1.15	0.87	1.53	0.331
**Mother’s education**												
Secondary or higher					1.00				1.00			
Primary					1.28	0.95	1.72	0.104	1.26	0.96	1.65	0.089
No education					1.66	1.24	2.23	0.001	1.65	1.29	2.13	<0.001
**Maternal age at child’s birth**												
less than 20					1.00				1.00			
20–29					0.82	0.69	0.99	0.034	0.90	0.75	1.08	0.242
30–39					0.76	0.58	0.99	0.040	0.92	0.70	1.21	0.561
40+					0.56	0.35	0.90	0.016	0.81	0.48	1.37	0.429
**Mother’s age**												
15–24					1.00				1.00			
25–34					1.16	0.99	1.37	0.073	0.99	0.85	1.15	0.878
35–49					1.64	1.17	2.30	0.004	1.18	0.84	1.65	0.339
**Mother’s marital status**												
Currently married					1.00				1.00			
Formerly married ^$^					0.48	0.21	1.09	0.079	0.37	0.15	0.92	0.032
**Maternal height**												
≥160 cm					1.00				1.00			
155–159					1.09	0.83	1.42	0.549	1.10	0.82	1.48	0.531
150–154					1.55	1.19	2.03	0.001	1.54	1.15	2.06	0.004
145–149					1.92	1.49	2.47	<0.001	2.00	1.51	2.65	<0.001
<145 cm					2.69	1.95	3.72	<0.001	2.98	2.14	4.15	<0.001
**Maternal BMI (kg/m^2^)**												
25+					1.00				1.00			
19–25					1.10	0.89	1.37	0.373	1.09	0.89	1.34	0.403
<= 18.5					1.29	1.00	1.68	0.053	1.25	0.97	1.61	0.088
**Combined birth rank and birth interval**												
1st birth rank					1.00				1.00			
2nd/3rd birth rank, more than 2 years interval					0.89	0.74	1.07	0.220	0.98	0.82	1.16	0.786
2nd/3rd birth rank, less than or equal 2					1.19	0.99	1.44	0.071	1.24	1.02	1.51	0.031
4th birth rank, more than 2 years interval					0.91	0.69	1.18	0.469	1.00	0.79	1.28	0.984
4th birth rank, less than or equal to 2					1.19	0.92	1.55	0.182	1.27	0.98	1.65	0.072
**Paternal occupation**												
Non-agriculture					1.00				1.00			
Agriculture					1.11	0.83	1.49	0.473	1.09	0.82	1.45	0.552
Not working					1.00	0.75	1.33	0.980	0.94	0.68	1.29	0.688
**Power over earnings (Woman has money autonomy)**												
By Husband alone					1.00				1.00			
Woman alone or joint decision					1.12	0.82	1.55	0.471	1.10	0.79	1.51	0.579
**Power over Household decision making**												
By Husband alone					1.00				1.00			
Woman alone or joint decision					0.86	0.63	1.17	0.329	0.89	0.62	1.27	0.529
**Woman has health care autonomy**												
By Husband alone					1.00				1.00			
Woman alone or joint decision					0.95	0.70	1.28	0.717	0.90	0.65	1.24	0.526
**Pooled Household wealth index**												
Richest					1.00				1.00			
Richer					0.93	0.74	1.18	0.567	0.99	0.78	1.25	0.910
Middle					1.05	0.82	1.33	0.714	1.07	0.85	1.37	0.558
Poorer					1.05	0.77	1.42	0.773	1.05	0.77	1.44	0.744
Poorest					1.11	0.81	1.52	0.515	1.12	0.82	1.52	0.487
**Source of drinking water**												
Not improved					1.00				1.00			
Improved					1.14	0.96	1.36	0.130	1.05	0.89	1.24	0.555
**Type of toilet facility**												
Improved					1.00				1.00			
Unimproved					1.04	0.85	1.26	0.725	1.11	0.92	1.33	0.273
**Combined mode and place of delivery**												
Caesarean and Health Facility					1.00				1.00			
Vaginal and Health Facility					1.52	1.19	1.94	0.001	1.61	1.30	2.00	<0.001
Home					1.69	1.31	2.18	<0.001	1.81	1.42	2.31	<0.001
**Delivery Assistance**												
Health professional					1.00				1.00			
Traditional birth attendant					0.81	0.66	1.00	0.050	0.79	0.65	0.97	0.021
Other untrained ^&^					0.93	0.80	1.09	0.385	0.92	0.79	1.08	0.316
**Antenatal clinic visits**												
≥8					1.00				1.00			
4 to 7					1.03	0.87	1.23	0.718	1.00	0.83	1.20	0.974
1 to 3					1.32	1.04	1.67	0.021	1.21	0.99	1.50	0.070
None					1.38	1.15	1.65	<0.001	1.16	0.97	1.41	0.102
**Media**												
**Reads newspaper**												
Not all					1.00				1.00			
Yes ^++^					1.10	0.91	1.33	0.323	1.11	0.93	1.33	0.250
**Listening radio**												
Not all					1.00				1.00			
Yes ^++^					0.95	0.78	1.16	0.633	1.01	0.82	1.25	0.891
**Watches television**												
Not all					1.00				1.00			
Yes ^++^					1.05	0.91	1.22	0.505	1.00	0.87	1.14	0.957
**Dietary diversity score**												
<4 food Inadequate									1.00			
4+ foods Adequate									0.80	0.66	0.96	0.015
**Initiation of breastfeeding**												
more than 1 h									1.00			
within 1 h									1.01	0.77	1.32	0.953
**Currently breastfeeding**												
Yes									1.00			
No									0.91	0.80	1.04	0.174
**Duration of breastfeeding**												
Up to 12 months									1.00			
>12 months									1.37	1.06	1.75	0.015
**Vitamin A supplement**												
Yes									1.00			
No									1.14	1.00	1.30	0.055
**Child’s age in months**												
0 to 5									1.00			
6 to 11									1.21	0.97	1.51	0.090
12 to 17									2.32	1.61	3.36	<0.001
18 to 23									3.51	2.38	5.17	<0.001
**Sex of child**												
Female									1.00			
Male									1.22	1.08	1.39	0.002
**Vaccination**												
No									1.00			
Yes **									1.04	0.88	1.23	0.670
**Had diarrhoea recently**												
No									1.00			
Yes									1.10	0.90	1.33	0.352
**Had fever in last two weeks**												
No									1.00			
Yes									0.95	0.79	1.14	0.609

$ = formerly in union/living with a man, never in union [includes married gauna]; &—assistance from friends, relatives, neighbours, no one and others; ++ = less than once a week and at least once a week; ** Yes if the child received a Bacillus Calmette–Guerin vaccination against tuberculosis; 3 doses of diphtheria, pertussis, and tetanus vaccine; ≥3 doses of polio vaccine; and 1 dose of measles vaccine and no otherwise.

**Table 2:** Factors associated with stunting in children aged 24–59 months.

Variables	Model 1: Basic				Model 2: Basic and Underlying				Model 3: Basic, Underlying and Immediate			
	OR	95% CI		*p*-Value	OR	95% CI		*p*-Value	OR	95% CI		*p*-Value
**Countries**												
Maldives	1.00				1.00				1.00			
India	3.73	2.63	5.30	<0.001	2.65	1.74	4.02	<0.001	2.69	1.77	4.08	<0.001
Bangladesh	3.60	2.34	5.54	<0.001	2.55	1.37	4.75	0.003	2.45	1.30	4.62	0.006
Nepal	4.32	2.97	6.31	<0.001	2.61	1.71	3.96	<0.001	2.62	1.71	4.02	<0.001
Pakistan	3.79	2.54	5.64	<0.001	3.37	2.17	5.23	<0.001	3.28	2.09	5.15	<0.001
**Type of place of residence**												
Urban	1.00				1.00				1.00			
Rural	1.86	1.62	2.14	<0.001	1.18	1.04	1.33	0.008	1.17	1.04	1.31	0.011
**Working status**												
Not working					1.00				1.00			
Working					0.87	0.69	1.08	0.199	0.86	0.69	1.08	0.196
**Mother’s education**												
Secondary or higher					1.00				1.00			
Primary					1.19	0.96	1.47	0.120	1.18	0.95	1.46	0.141
No education					1.47	1.28	1.70	<0.001	1.46	1.27	1.69	<0.001
**Maternal age at child’s birth**												
Less than 20					1.00				1.00			
20–29					0.98	0.81	1.18	0.812	0.98	0.81	1.18	0.810
30–39					1.40	0.57	3.42	0.460	1.41	0.58	3.46	0.448
40+					1.45	0.54	3.90	0.458	1.44	0.53	3.88	0.472
**Mother’s age**												
15–24					1.00				1.00			
25–34					0.89	0.77	1.03	0.119	0.89	0.77	1.03	0.118
35–49					0.57	0.25	1.31	0.189	0.57	0.25	1.31	0.187
**Mother’s marital status**												
Currently married					1.00				1.00			
Formerly married ^$^					1.18	0.86	1.62	0.306	1.17	0.85	1.61	0.335
**Maternal height**												
≥160 cm					1.00				1.00			
155–159					1.55	1.24	1.94	<0.001	1.53	1.22	1.91	<0.001
150–154					2.40	1.99	2.90	<0.001	2.38	1.97	2.87	<0.001
145–149					3.68	2.91	4.64	<0.001	3.63	2.87	4.60	<0.001
<145 cm					4.81	3.75	6.18	<0.001	4.76	3.71	6.11	<0.001
**Maternal BMI (kg/m^2^)**												
25+					1.00				1.00			
19–25					1.08	0.86	1.37	0.498	1.08	0.85	1.37	0.529
<=18.5					1.43	1.17	1.74	<0.001	1.42	1.16	1.73	0.001
**Combined birth rank and birth interval**												
1st birth rank					1.00				1.00			
2nd/3rd birth rank, more than 2 years interval					1.36	1.16	1.61	<0.001	1.37	1.16	1.61	<0.001
2nd/3rd birth rank, less than or equal 2					1.95	1.61	2.35	<0.001	1.95	1.61	2.36	<0.001
4th birth rank, more than 2 years interval					1.70	1.37	2.12	<0.001	1.70	1.37	2.11	<0.001
4th birth rank, less than or equal to 2					1.63	1.23	2.15	0.001	1.56	1.17	2.07	0.002
**Paternal occupation**												
Non-agriculture					1.00				1.00			
Agriculture					1.13	0.95	1.34	0.174	1.14	0.96	1.35	0.146
Not working					1.08	0.89	1.32	0.412	1.09	0.90	1.32	0.401
**Power over earnings (Woman has money autonomy)**												
By Husband alone					1.00				1.00			
Woman alone or joint decision					1.27	1.01	1.59	0.043	1.28	1.02	1.60	0.035
**Power over Household decision making**												
By Husband alone					1.00				1.00			
Woman alone or joint decision					0.85	0.63	1.14	0.287	0.83	0.62	1.12	0.221
**Woman has health care autonomy**												
By Husband alone					1.00				1.00			
Woman alone or joint decision					0.91	0.70	1.20	0.512	0.93	0.71	1.21	0.572
**Pooled Household wealth index**												
Richest					1.00				1.00			
Richer					1.03	0.79	1.35	0.805	1.03	0.79	1.35	0.813
Middle					1.37	1.13	1.66	0.002	1.36	1.12	1.66	0.002
Poorer					1.43	1.18	1.75	<0.001	1.43	1.17	1.74	<0.001
Poorest					1.65	1.31	2.06	<0.001	1.64	1.31	2.05	<0.001
**Source of drinking water**												
Not improved					1.00				1.00			
Improved					1.02	0.90	1.14	0.790	1.02	0.90	1.15	0.744
**Type of toilet facility**												
Improved					1.00				1.00			
Unimproved					1.00	0.86	1.16	0.950	1.00	0.86	1.15	0.963
**Combined mode and place of delivery**												
Caesarean and Health Facility					1.00				1.00			
Non and Health Facility					1.27	0.97	1.67	0.085	1.27	0.97	1.67	0.086
Non C and Home					1.21	0.91	1.62	0.193	1.23	0.92	1.64	0.171
**Delivery Assistance**												
Health professional					1.00				1.00			
Traditional birth attendant					1.03	0.90	1.17	0.674	1.02	0.90	1.16	0.735
Other untrained ^&^					1.12	0.96	1.31	0.159	1.10	0.94	1.28	0.242
**Antenatal clinic visits**												
≥8					1.00				1.00			
4 to 7					1.09	0.86	1.39	0.482	1.09	0.86	1.39	0.479
1 to 3					1.41	1.05	1.88	0.021	1.41	1.05	1.88	0.022
None					1.17	0.95	1.45	0.141	1.20	0.97	1.49	0.096
**Media**												
**Reads newspaper**												
Not all					1.00				1.00			
Yes ^++^					1.18	0.98	1.43	0.085	1.18	0.98	1.43	0.087
**Listening radio**												
Not all					1.00				1.00			
Yes ^++^					1.23	0.96	1.57	0.107	1.22	0.95	1.58	0.120
**Watches television**												
Not all					1.00				1.00			
Yes ^++^					0.86	0.76	0.97	0.012	0.86	0.76	0.97	0.015
**Dietary diversity score**												
<4 food Inadequate									1.00			
4+ foods Adequate									0.88	0.71	1.11	0.284
**Vitamin A supplement**												
Yes									1.00			
No									1.01	0.92	1.10	0.837
**Sex of child**												
Female									1.00			
Male									0.96	0.87	1.07	0.481
**Vaccination**												
No									1.00			
Yes **									0.99	0.86	1.13	0.835
**Had diarrhoea recently**												
No									1.00			
Yes									1.21	1.03	1.42	0.018
**Had fever in last two weeks**												
No									1.00			
Yes									1.16	1.02	1.31	0.022

$ = formerly in union/living with a man, never in union [includes married gauna]; &—assistance from friends, relatives, neighbours, no one and others; ++ = less than once a week and at least once a week; ** Yes if the child received a Bacillus Calmette–Guerin vaccination against tuberculosis; 3 doses of diphtheria, pertussis, and tetanus vaccine; ≥3 doses of polio vaccine; and 1 dose of measles vaccine and No otherwise.

**Table 3:** Factors associated with stunting in children aged 0–59 months.

Variables	Model 1: Basic				Model 2: Basic and Underlying				Model 3: Basic, Underlying and Immediate			
	OR	95% CI		*p*-Value	OR	95% CI		*p*-Value	OR	95% CI		*p*-Value
**Countries**												
Maldives	1.00				1.00				1.00			
India	2.78	1.97	3.93	<0.001	2.29	1.58	3.32	<0.001	2.07	1.44	2.98	<0.001
Bangladesh	2.61	1.81	3.76	<0.001	1.85	1.20	2.87	0.006	1.77	1.16	2.71	0.008
Nepal	2.96	2.06	4.25	<0.001	1.91	1.36	2.70	<0.001	1.77	1.25	2.51	0.001
Pakistan	2.61	1.77	3.85	<0.001	2.47	1.71	3.57	<0.001	2.28	1.57	3.31	<0.001
**Type of place of residence**												
Urban	1.00				1.00				1.00			
Rural	1.64	1.45	1.85	<0.001	1.13	1.01	1.25	0.028	1.11	1.00	1.23	0.056
**Working status**												
Not working					1.00				1.00			
Working					1.02	0.86	1.21	0.851	0.97	0.80	1.17	0.756
**Mother’s education**												
Secondary or higher					1.00				1.00			
Primary					1.21	0.97	1.53	0.095	1.21	0.97	1.52	0.096
No education					1.55	1.31	1.83	<0.001	1.59	1.34	1.88	<0.001
**Maternal age at child’s birth**												
Less than 20					1.00				1.00			
20–29					0.80	0.69	0.93	0.004	0.89	0.77	1.02	0.091
30–39					0.87	0.52	1.46	0.596	1.02	0.61	1.69	0.950
40+					0.81	0.44	1.51	0.513	0.99	0.54	1.80	0.967
**Mother’s age**												
15–24					1.00				1.00			
25–34					1.14	1.02	1.26	0.018	1.01	0.91	1.12	0.877
35–49					1.11	0.70	1.78	0.657	0.90	0.56	1.43	0.645
**Mother’s marital status**												
Currently married					1.00				1.00			
Formerly married ^$^					1.01	0.73	1.39	0.958	0.98	0.72	1.33	0.886
**Maternal height**												
≥160 cm					1.00				1.00			
155–159					1.36	1.14	1.64	0.001	1.36	1.12	1.64	0.002
150–154					2.03	1.73	2.38	<0.001	2.03	1.72	2.40	<0.001
145–149					2.88	2.38	3.49	<0.001	2.87	2.37	3.48	<0.001
<145 cm					3.73	2.95	4.72	<0.001	3.80	2.99	4.82	<0.001
**Maternal BMI (kg/m^2^)**												
25+					1.00				1.00			
19–25					1.06	0.85	1.32	0.615	1.09	0.89	1.34	0.405
<= 18.5					1.31	1.08	1.58	0.005	1.36	1.14	1.63	0.001
**Combined birth rank and birth interval**												
1st birth rank					1.00				1.00			
2nd/3rd birth rank, more than 2 years interval					1.16	1.05	1.27	0.003	1.19	1.07	1.31	0.001
2nd/3rd birth rank, less than or equal 2					1.62	1.12	1.57	<0.001	1.67	1.43	1.94	<0.001
4th birth rank, more than 2 yrs interval					1.32	1.12	1.57	0.001	1.42	1.21	1.66	<0.001
4th birth rank, less than or equal to 2					1.44	1.17	1.76	<0.001	1.50	1.23	1.84	<0.001
**Paternal occupation**												
Non-agriculture					1.00				1.00			
Agriculture					1.13	0.97	1.32	0.114	1.14	0.97	1.33	0.101
Not working					1.09	0.95	1.26	0.222	1.07	0.93	1.23	0.343
**Power over earnings (Woman has money autonomy)**												
By Husband alone					1.00				1.00			
Woman alone or joint decision					1.23	1.01	1.49	0.036	1.22	1.00	1.49	0.053
**Power over Household decision making**												
By Husband alone					1.00				1.00			
Woman alone or joint decision					0.87	0.70	1.07	0.185	0.85	0.69	1.06	0.151
**Woman has health care autonomy**												
By Husband alone					1.00				1.00			
Woman alone or joint decision					0.92	0.74	1.13	0.412	0.92	0.74	1.13	0.416
**Pooled Household wealth index**												
Richest					1.00				1.00			
Richer					0.99	0.78	1.24	0.899	0.99	0.78	1.24	0.899
Middle					1.22	1.02	1.46	0.026	1.22	1.02	1.45	0.033
Poorer					1.25	1.03	1.53	0.026	1.24	1.01	1.52	0.036
Poorest					1.40	1.13	1.72	0.002	1.39	1.13	1.73	0.002
**Source of drinking water**												
Not improved					1.00				1.00			
Improved					1.08	0.98	1.20	0.127	1.04	0.93	1.16	0.521
**Type of toilet facility**												
Improved					1.00				1.00			
Unimproved					0.99	0.85	1.16	0.902	1.02	0.88	1.18	0.839
**Combined mode and place of delivery**												
Caesarean and Health Facility					1.00				1.00			
Non and Health Facility					1.39	1.08	1.80	0.011	1.37	1.08	1.75	0.011
Non C and Home					1.43	1.10	1.86	0.008	1.43	1.11	1.84	0.006
**Delivery Assistance**												
Health professional					1.00				1.00			
Traditional birth attendant					0.94	0.83	1.06	0.313	0.94	0.83	1.06	0.321
Other untrained ^&^					1.04	0.93	1.16	0.468	1.04	0.93	1.17	0.446
**Antenatal clinic visits**												
≥8					1.00				1.00			
4 to 7					1.09	0.91	1.31	0.350	1.07	0.90	1.28	0.432
1 to 3					1.34	1.08	1.66	0.008	1.35	1.09	1.67	0.005
None					1.33	1.16	1.53	<0.001	1.22	1.05	1.42	0.008
**Media**												
**Reads newspaper**												
Not all					1.00				1.00			
Yes ^++^					1.15	0.97	1.37	0.106	1.15	0.97	1.36	0.111
**Listening radio**												
Not all					1.00				1.00			
Yes ^++^					1.14	0.90	1.44	0.264	1.13	0.90	1.41	0.286
**Watches television**												
Not all					1.00				1.00			
Yes ^++^					0.95	0.85	1.06	0.323	0.92	0.82	1.03	0.131
**Dietary diversity score**												
<4 food Inadequate									1.00			
4+ foods Adequate									1.03	0.88	1.19	0.741
**Vitamin A supplement**												
Yes									1.00			
No									0.94	0.86	1.02	0.117
**Child age in months**												
0–23 months									1.00			
24–59 months									1.40	1.26	1.56	<0.001
**Sex of child**												
Female									1.00			
Male									1.05	0.96	1.14	0.302
**Vaccination**												
No									1.00			
Yes **									1.23	1.10	1.37	<0.001
**Had diarrhoea recently**												
No									1.00			
Yes									1.12	0.99	1.27	0.060
**Had fever in last two weeks**												
No									1.00			
Yes									1.06	0.96	1.17	0.240

$ = formerly in union/living with a man, never in union [includes married gauna]; &—assistance from friends, relatives, neighbours, no one and others; ++ = less than once a week and at least once a week; ** Yes if the child received a Bacillus Calmette–Guerin vaccination against tuberculosis; 3 doses of diphtheria, pertussis, and tetanus vaccine; ≥3 doses of polio vaccine; and 1 dose of measles vaccine and No otherwise.

## Supplementary Materials

The following are available online at https://www.mdpi.com/2072-6643/12/12/3875/s1, Material 1: Characteristics of parents and children aged 0–59 months; Material 2: Country-specific data.
