# Supplementary material for: Factors Associated with Stunting among Children under 5 Years in Five South Asian Countries (2014–2018): Analysis of Demographic Health Surveys
Source: Nutrients. 2020 Dec 18;12(12):3875. doi: 10.3390/nu12123875 (PMC7767090; doi:10.3390/nu12123875)
Supplement: Supplementary file 1 [file nutrients-12-03875-s001.zip › nutrients-988838-supplementary/nutrients-988838-supplementary material 1.docx]

**Supplementary material 1**

**Table.** Characteristics of parents and children aged 0–59 months in five South Asian countries 2014–2018.

|  | *N* (%) | % | *N** | %* |
| --- | --- | --- | --- | --- |
| Immediate Factors |  |  |  |  |
| Dietary diversity score |  |  |  |  |
| <4 food Inadequate | 502,720 | 89.05 | 261,538 | 90.75 |
| 4+ foods Adequate | 61,799 | 10.95 | 26,662 | 9.25 |
| Initiation of breastfeeding ^^ |  |  |  |  |
| More than 1 h | 125,655 | 57.55 | 66,756 | 58.98 |
| Within 1 h | 92,689 | 42.45 | 46,435 | 41.02 |
| Currently breastfeeding ^^ |  |  |  |  |
| Yes | 180,997 | 82.9 | 95,547 | 84.41 |
| No | 37,347 | 17.1 | 17,644 | 15.59 |
| Duration of breastfeeding ^^ |  |  |  |  |
| Up to 12 months | 132,752 | 62.54 | 69,625 | 63.56 |
| >12 months | 79,518 | 37.46 | 39,916 | 36.44 |
| Had diarrhoea recently |  |  |  |  |
| No | 497,294 | 91.63 | 248,960 | 90.73 |
| Yes | 45,449 | 8.37 | 25,432 | 9.27 |
| Had fever in last two weeks |  |  |  |  |
| No | 489,556 | 86.99 | 246,288 | 85.76 |
| Yes | 73,202 | 13.01 | 40,904 | 14.24 |
| Vitamin A supplement |  |  |  |  |
| Yes | 330,858 | 61.50 | 146,375 | 54.00 |
| No | 207,088 | 38.50 | 124,667 | 46.00 |
| Vaccination |  |  |  |  |
| No | 305,637 | 54.14 | 163,223 | 56.64 |
| Yes ** | 258,882 | 45.86 | 124,977 | 43.36 |
| Child’s age in months |  |  |  |  |
| 0 to 5 | 46,475 | 8.2 | 26,459 | 9.2 |
| 6 to 11 | 57,785 | 10.2 | 29,444 | 10.2 |
| 12 to 17 | 57,219 | 10.1 | 29,097 | 10.1 |
| 18 to 23 | 56,863 | 10.1 | 28,191 | 9.8 |
| 24 to 29 | 59,833 | 10.6 | 28,712 | 10.0 |
| 30 to 35 | 54,883 | 9.7 | 28,490 | 9.9 |
| 36 to 41 | 61,009 | 10.8 | 30,288 | 10.5 |
| 42 to 47 | 56,638 | 10.0 | 29,601 | 10.3 |
| 48 to 53 | 58,309 | 10.3 | 28,799 | 10.0 |
| 54 to 59 | 55,503 | 9.8 | 29,119 | 10.1 |
| Sex of child |  |  |  |  |
| Male | 298,109 | 52.81 | 149,820 | 51.98 |
| Female | 266,409 | 47.19 | 138,380 | 48.02 |
| UNDERLYING FACTORS |  |  |  |  |
| *Mother’s characteristics* |  |  |  |  |
| Mother’s age |  |  |  |  |
| 15–24 | 203,772 | 36.10 | 94,191 | 32.68 |
| 25–34 | 315,204 | 55.84 | 163,376 | 56.69 |
| 35–49 | 45,543 | 8.07 | 30,633 | 10.63 |
| Maternal age at child’s birth |  |  |  |  |
| Less than 20 | 85,396 | 15.13 | 36,792 | 12.77 |
| 20–29 | 404,288 | 71.62 | 202,997 | 70.44 |
| 30–39 | 71,155 | 12.60 | 44,975 | 15.61 |
| 40+ | 3679 | 0.65 | 3436 | 1.19 |
| Maternal height |  |  |  |  |
| ≥160 cm | 39,546 | 7.42 | 21,122 | 7.70 |
| 155–159 | 104,417 | 19.59 | 54,914 | 20.01 |
| 150–154. | 179,901 | 33.75 | 93,376 | 34.03 |
| 145–149 | 141,057 | 26.46 | 73,505 | 26.79 |
| <145 cm | 68,079 | 12.77 | 31,462 | 11.47 |
| Maternal BMI (kg/m^2^) |  |  |  |  |
| 25+ | 89,949 | 16.90 | 48,632 | 17.75 |
| 18–25 | 341,610 | 64.17 | 184,644 | 67.38 |
| ≤18.5 | 100,822 | 18.94 | 40,768 | 14.88 |
| Mother’s marital status |  |  |  |  |
| Currently married | 557,795 | 98.89 | 283,875 | 98.63 |
| Formerly married ^$^ | 6246 | 1.11 | 3934 | 1.37 |
| Birth order |  |  |  |  |
| 1 | 234,944 | 41.6 | 105,439 | 36.6 |
| 2–4 | 298,296 | 52.8 | 157,198 | 54.5 |
| ≥5 | 31,278 | 5.5 | 25,563 | 8.9 |
| Birth interval (preceding) |  |  |  |  |
| no previous birth | 236,359 | 41.9 | 106,197 | 36.9 |
| <24 months | 87,271 | 15.5 | 47,999 | 16.7 |
| >24 months | 240,798 | 42.7 | 133,924 | 46.5 |
| Combined birth rank and birth interval | | | | |
| 1st birth rank | 234,944 | 41.62 | 105,439 | 36.59 |
| 2nd/3rd birth rank, >2 years | 209,159 | 37.05 | 110,447 | 38.32 |
| 2nd/3rd birth rank, ≤2 | 89,137 | 15.79 | 46,751 | 16.22 |
| 4th birth rank, >interval | 21,728 | 3.85 | 18,033 | 6.26 |
| 4th birth rank, ≤2 | 9550 | 1.69 | 7530 | 2.61 |
| Working status |  |  |  |  |
| Not working | 539,465 | 95.56 | 273,808 | 95.01 |
| Working | 25,053 | 4.44 | 14,389 | 4.99 |
| Mother’s education |  |  |  |  |
| Secondary or higher | 132,099 | 23.40 | 90,502 | 31.40 |
| Primary | 81,114 | 14.37 | 43,428 | 15.07 |
| No education | 351,305 | 62.23 | 154,270 | 53.53 |
| Paternal occupation |  |  |  |  |
| Non-agriculture | 96,051 | 17.00 | 50,218 | 17.43 |
| Agriculture | 30,003 | 5.30 | 19,651 | 6.82 |
| Not working | 438,244 | 77.70 | 218,182 | 75.74 |
| Power over earnings (Woman has money autonomy) | | | | |
| By Husband alone | 494,969 | 87.68 | 248,523 | 86.2 |
| Woman alone or joint decision | 69,549 | 12.32 | 39,677 | 13.8 |
| Power over Household decision making | | | | |
| By Husband alone | 464,605 | 82.3 | 233,624 | 81.1 |
| Woman alone or joint decision | 99,913 | 17.7 | 54,576 | 18.9 |
| Woman has health care autonomy |  |  |  |  |
| By Husband alone | 477,134 | 84.5 | 240,663 | 83.5 |
| Woman alone or joint decision | 87,384 | 15.5 | 47,537 | 16.5 |
| *Household* |  |  |  |  |
| Pooled Household wealth index |  |  |  |  |
| Poorest | 78,060 | 13.8 | 57,642 | 20.0 |
| Poorer | 83,593 | 14.8 | 57,641 | 20.0 |
| Middle | 99,919 | 17.7 | 57,637 | 20.0 |
| Richer | 140,627 | 24.9 | 57,641 | 20.0 |
| Richest | 162,320 | 28.8 | 57,639 | 20.0 |
| Source of drinking water |  |  |  |  |
| Not improved | 58,376 | 10.3 | 42,551 | 14.8 |
| Improved | 506,142 | 89.7 | 245,649 | 85.2 |
| Type of toilet facility |  |  |  |  |
| Improved | 319,836 | 56.7 | 142,679 | 49.5 |
| Unimproved | 244,603 | 43.3 | 145,453 | 50.5 |
| *Access to services* |  |  |  |  |
| *Healthcare utilisation factors* |  |  |  |  |
| Place of delivery |  |  |  |  |
| Home ^ | 101,719 | 18.2 | 73,821 | 25.9 |
| Health Facility | 457,175 | 81.8 | 211,213 | 74.1 |
| Mode of delivery |  |  |  |  |
| Non-caesarean | 421,582 | 75.5 | 244,091 | 85.7 |
| Caesarean section | 136,657 | 24.5 | 40,674 | 14.3 |
| Combined mode and place of delivery |  |  |  |  |
| Caesarean and Health Facility | 101,179 | 18.1 | 73,645 | 25.9 |
| Vaginal and Health Facility | 320,398 | 57.4 | 170,442 | 59.9 |
| Home | 136,656 | 24.5 | 40,672 | 14.3 |
| Delivery Assistance |  |  |  |  |
| Health professional | 404,219 | 72.7 | 185,946 | 65.8 |
| Traditional birth attendant | 48,560 | 8.7 | 34,565 | 12.2 |
| Other untrained ^&^ | 103,133 | 18.6 | 62,303 | 22.0 |
| Antenatal clinic visits |  |  |  |  |
| ≥8 | 87,381 | 16.28 | 24,789 | 8.87 |
| 4 to 7 | 139,816 | 26.05 | 66,440 | 23.77 |
| 1 to 3 | 110,784 | 20.64 | 71,963 | 25.74 |
| None | 198,638 | 37.02 | 116,360 | 41.62 |
| *Media* |  |  |  |  |
| Reads newspaper |  |  |  |  |
| Not all | 357,336 | 63.30 | 201,210 | 69.82 |
| Yes ^++^ | 207,160 | 36.70 | 86,966 | 30.18 |
| Listening radio |  |  |  |  |
| Not all | 481,048 | 85.21 | 244,719 | 84.91 |
| Yes ^++^ | 83,471 | 14.79 | 43,481 | 15.09 |
| Watches television |  |  |  |  |
| Not all | 136,315 | 24.15 | 95,183 | 33.03 |
| Yes ^++^ | 428,203 | 75.85 | 193,017 | 66.97 |
| BASIC FACTORS |  |  |  |  |
| Countries |  |  |  |  |
| Maldives | 14,853 | 2.63 | 7886 | 2.7 |
| India | 519,243 | 92.0 | 259,627 | 90.1 |
| Bangladesh | 4708 | 0.8 | 3085 | 1.1 |
| Nepal | 6739 | 1.2 | 4994 | 1.7 |
| Pakistan | 18,975 | 3.4 | 12,608 | 4.4 |
| Type of place of residence |  |  |  |  |
| Urban | 251,655 | 44.6 | 72,552 | 25.2 |
| Rural | 312,863 | 55.4 | 215,648 | 74.8 |

^^ analysis was restricted to 0–23 months. ^ = 176 observations were excluded because of the missing mode of delivery. *N** = weighted count, %* = weighted percent; % = unweighted percent. $ = formerly in union/living with a man, never in union [includes married gauna]; &—assistance from friends, relatives, neighbours, no one and others; ++ = less than once a week and at least once a week; ** Yes if the child received a Bacillus Calmette–Guerin vaccination against tuberculosis; 3 doses of diphtheria, pertussis, and tetanus vaccine; ≥3 doses of polio vaccine; and 1 dose of measles vaccine and No otherwise.
